# Quality Characterization of Honeys from Iraqi Kurdistan and Comparison with Central European Honeys

**DOI:** 10.3390/foods13142228

**Published:** 2024-07-16

**Authors:** Matej Tkáč, Fouad Ali Abdullah Abdullah, Lenka Vorlová, Klára Bartáková, Šárka Bursová, Zdeňka Javůrková

**Affiliations:** 1 Department of Animal Origin Food & Gastronomic Sciences, Faculty of Veterinary Hygiene and Ecology, University of Veterinary Sciences Brno, Palackého třída 1946/1, 612 42 Brno, Czech Republic; abdullahf@vfu.cz (F.A.A.A.); vorloval@vfu.cz (L.V.); bartakovak@vfu.cz (K.B.); bursovas@vfu.cz (Š.B.); 2 Department of Plant Origin Food Sciences, Faculty of Veterinary Hygiene and Ecology, University of Veterinary Sciences Brno, Palackého třída 1946/1, 612 42 Brno, Czech Republic; javurkovaz@vfu.cz

**Keywords:** Dohuk Governorate, physicochemical parameters, hydroxymethylfurfural, color intensity, phenolic content, diastase activity, DPPH, FRAP

## Abstract

The main objective of this work was to determine the significant physicochemical and bioactive parameters of honeys originating from Iraqi Kurdistan. For this purpose, honey samples (*n* = 16) were taken directly from Kurdish beekeepers from different places of Dohuk and Erbil Governorate in 2018 and 2022. The following parameters were analyzed: moisture content, electrical conductivity, diastase activity, hydroxymethylfurfural content, pH, free acidity, content of saccharides, total phenolic content, total flavonoid content, color, color intensity and DPPH, FRAP. Another objective of this work was to compare the properties of Kurdistan honeys with the requirements of European Union law and with the honeys of Central Europe. A total of 48 honey samples were included in the comparison. It was detected that the honeys produced in Iraqi Kurdistan met the strict quality criteria set by European law, which is important information for local beekeepers and experts in relation to honey production and an opportunity to set local limits of honey quality. Despite the different climatic and topographical conditions, the available flora, and the different species of bees, the properties of Iraqi Kurdistan honeys were similar to the properties of honeys from the Czech and Slovak Republics.

## 1. Introduction

In Europe, beekeeping and honey production are a common part of agriculture, taking into consideration the appropriate climatic, environmental, and economic conditions. According to Al-Badri [1], the honey production in Iraq is influenced by environmental issues like little rain, desertification, use of pesticides, and economic problems. Iraq is climatically diverse, with continental, subtropical semi-arid type, and Mediterranean climate in the north and northeastern regions. Rainfall is very seasonal. Summers in Iraq are characterized by drought, hot to extremely hot, with temperatures of over 43 degrees Celsius during July and August [2]. The environment of Kurdistan is very diverse and consists of areas of forests and mountain herbs (mainly with oaks, almonds, walnuts, pines, pistachios, grasses, and shrubs), plains regions (with grasses, bulbous, thorny, and spiny plants), and areas of riverbanks (with willows, shrubs, grasses, liquorice, and thorny plants). This diverse flora provides pasture for the bees. In 2008, 3258 beekeepers were recorded in Dohuk Governorate of Iraqi Kurdistan, which is the second highest number after Sulaimania Governorate. Total annual production of honey in Iraqi Kurdistan ranges from 600 to 800 tons [3].

To date, no works related to the analysis of honey from the Dohuk Governorate of Iraqi Kurdistan have been published and a narrow range of work has been published about the analysis of honeys originating from the Kurdistan region of Iraq [4,5] and Kurdistan region of Iran [6,7,8]. Some works are focused specifically on the biological activity of Iraqi honey and scarce data have been published on the physicochemical properties. Ali et al. [9] identified by the GC-MS method in Iraqi honeys a wide range of volatile compounds like alcohols, phenols, ketones, organic acids, and esters with antibacterial, antifungal, and anticancer activity. Hamed et al. [10] focused on the determination of water-soluble vitamins of the B-complex group in Iraqi honeys by the HPLC method. Al-Hasani et al. [11] detected an antibacterial effect of Iraqi honey samples against Gram-positive and Gram-negative bacteria at a concentration of 100% and the inhibition effect of honey decreased as honey diluted. Abu-Almaaly [12] focused on the identification of some heavy metals in samples of Iraqi honeys by the atomic absorption technique. The same author detected cadmium, lead, nickel, and iron in honey samples, and concentrations of these heavy metals varied according to the areas of honey collection.

The chemical composition of honey depends on the floral source and geographical origin. Honey is a complex substance of different components such as carbohydrates, water, proteins, amino acids, enzymes, vitamins, phenolic acids, minerals, volatile compounds, pigments, and other substances [13].

From a geographical point of view, compared to the diverse climate of Iraq, the climate of the Czech and Slovak Republics is continental with warm, dry summers and cold winters. During summer (from June to September), daytime temperatures vary between 20 and 25 °C, with some hot days with temperatures above 30 °C. Topographically, the territory of the Czech Republic is represented by plains, hills, and plateaus, some surrounded by low mountains, and the territory of the Slovak Republic is represented by lowlands in the south, and mountains in the central and northern parts of the country. The predominance of arable land over permanent green areas is typical for both countries [14]. In 2022, arable land in Slovakia represented 71.5%, permanent green areas 27.5%, and vineyards, orchards, and gardens about 0.9%. From the point of view of the use of arable land, cereals (55%) and oilseeds like rapeseed, sunflower, and soybean (22%) dominated [15]. The main crops grown in the Czech Republic are cereals, rapeseed, potatoes, sugar beet, corn, and others [14].

In addition to the climatic and topographic differences between Iraq and the Czech and Slovak Republics, there is a difference in the species of bees. *Apis mellifera* L. is the most widespread species of bee for producing honey and other bee products in the Czech and Slovak Republics [16]. According to Ruttner [17], the distribution of two races of *Apis mellifera* bees, namely *Apis mellifera carnica* and *Apis mellifera mellifera*, affects the territory of the Czech and Slovak Republics. The assessment of wing morphometry of bees originating from the Slovak Republic met the standards for *Apis mellifera carnica* [18]. On the other hand, in Iraqi Kurdistan is widespread another subspecies of *Apis mellifera*, specifically *Apis mellifera meda* [19] and wild honeybees can also be found there.

In the European Union, the quality of honey is assessed by meeting the criteria specified in Directive of the European Union n. 110/2001. This directive specifies honey as a substance produced by bees of the species *Apis mellifera* and establishes, in addition to sensory requirements, physical and chemical requirements. Specifically, it specifies criteria for saccharide content, hydroxymethylfurfural content, moisture content, electrical conductivity, free acidity, diastase activity, and water-insoluble content in honeys [20]. In Kurdistan, there are no standard specifications for evaluating the quality of produced honey, and there are no specialized laboratories [3].

Data related to the honeys from Iranian Kurdistan were published, but due to the scarcity of published data related to the honeys of Iraqi Kurdistan, the main objectives were to (1) analyze specific physicochemical and bioactive parameters relating to honey quality and biological value in honeys collected directly from Kurdish beekeepers from Dohuk and Erbil Governorates of Iraqi Kurdistan, (2) to compare the quality of Kurdistan honeys with the requirements of European Union law, and (3) to compare the parameters of Kurdistan honeys with honeys directly from Czech and Slovak beekeepers.

## 2. Materials and Methods

### 2.1. Samples from Kurdish Beekeepers

A total of 16 honeys originating directly from Kurdish beekeepers from Iraqi Kurdistan were analyzed. Ten honey samples came from the production of 2018 and were labeled KH1-KH10. From this group of honeys, eight samples of honey came from beekeeping and two samples from wild bees. The other six honeys KH11-KH16 came from the 2022 production, and all came from beekeeping. Almost all the samples came from Dohuk Governorate; only one sample KH8 came from Erbil Governorate. Honey samples were taken in plastic laboratory sample boxes intended for contact with food and were stored at a temperature of 21 ± 2 °C, in the dark. A detailed specification of the analyzed honey samples from Kurdish beekeepers is given in Table 1. Information provided by beekeepers regarding breeding and available flora is provided in Table S1 (Supplementary Materials).

### 2.2. Samples from Czech and Slovak Beekeepers

To compare the properties of Kurdistan honeys with European honeys, samples of Czech and Slovak honeys were taken and analyzed. A total of 32 samples were analyzed; twenty-two samples came from Czech beekeepers, ten from the production of 2018 (CZ1-CZ10) and twelve from the production of 2022 (CZ11-CZ22). The remaining ten honey samples came from Slovak beekeepers from the production of 2018 (SK1-SK10). Honey samples were taken in plastic laboratory sample boxes intended for contact with food and were stored at a temperature of 21 ± 2 °C, in the dark. A detailed specification of the analyzed honey samples from Czech and Slovak beekeepers is provided in Table S2 (Supplementary Material).

When selecting all the samples for analysis (Kurdistan, Czech, and Slovak), the representativeness of the investigated set was ensured by random selection. Additional information is provided in Section 3.4.3 Limitation of Study.

### 2.3. Physicochemical Parameters

All chemicals used were of analytical grade. Diastase activity, hydroxymethylfurfural content, fructose, glucose and sucrose content, moisture content, electrical conductivity, pH, and free acidity were analyzed according to the Harmonized Methods of the International Honey Commission [21] with some modifications.

Diastase activity in honey samples was analyzed spectrophotometrically based on the Phadebas^®^ method (Phadebas^®^ Honey Diastase Test, Phadebas^®^ AB, Kristianstad, Sweden). The absorbance was measured on a spectrometer Specord 200Plus (Analytic Jena AG, Jena, Germany) at 620 nm. Based on the measured absorbance, diastase activity was calculated and expressed in diastase number, which correspond to the Schade scale.

The hydroxymethylfurfural concentration was determined using liquid chromatography with UV detection (285 nm). The analysis was carried out using an HPLC system consisting of separations module Alliance 2695 and Photodiode Array Detector 2996 (Waters, Milford, MA, USA). A Zorbax Eclipse XDB-C18-5 μm 4.6 × 150 mm column (Agilent, Santa Clara, CA, USA) was used for the separation. Isocratic elution was used, and the mobile phase consisted of a mixture of methanol and distilled water in a ratio of 10:90 (*v*/*v*). The flow rate of the mobile phase was 1.0 mL/min, 20 μL was injected into the column, and one run lasted 10 min. The chromatographic column was heated to a temperature of 35 °C. Concentration of hydroxymethylfurfural was quantified using the calibration curve (0.01–120 mg/L, R^2^ = 0.9980) of certified reference material of hydroxymethylfurfural (Sigma-Aldrich, St. Louis, MO, USA). Based on the applied dilution, the concentration of hydroxymethylfurfural was calculated and expressed in mg per kg of honey.

The concentrations of fructose, glucose, and sucrose were determined using liquid chromatography with refractometric detection. The analysis was carried out using an HPLC system consisting of separations module Alliance 2695 and Refractometric Detector 2414 (Waters, Milford, MA, USA). A X-Bridge Amide 3.5 μm, 4.6 × 150 mm column (Waters, Milford, MA, USA) was used for the separation. Isocratic elution was used, and the mobile phase consisted of a mixture of acetonitrile and distilled water in a ratio of 75:25 (*v*/*v*) and 0.2% triethylamine. The flow rate of the mobile phase was 0.8 mL/min, 10 μL was injected into the column, and one run lasted 15 min. The chromatographic column and detector were heated to a temperature of 45 °C. Concentration of saccharides was quantified using the calibration curves (fructose: 0.75–6.0 mg/L, R^2^ = 0.9999; glucose: 0.75–6.0 mg/L, R^2^ = 0.9999; sucrose: 0.06–3.0 mg/L, R^2^ = 0.9997) of the certified reference standards (Sigma-Aldrich, St. Louis, MO, USA). Based on the applied dilution, the concentrations of fructose, glucose, and sucrose were calculated and expressed in g per 100 g of honey.

The moisture content was determined by the optical method using a bench-top laboratory Abbé refractometer (AR4, A. Krüss Optronic GmbH, Hamburg, Germany). Based on the determined refractive index, after correction to a temperature of 20 °C, the moisture content was evaluated and expressed in g per 100 g of honey.

Electrical conductivity was determined electrometrically in a solution of honey containing 20 g of dry matter in 100 mL of distilled water. The inoLab Cond 730 conductometer (WTW, Weilheim, Germany) was used. After correcting the measured conductivities to a temperature of 20 °C, the electrical conductivities were converted to milli Siemens per meter (mS/m).

Free acidity and pH were determined using pH meter MP230 (Mettler-Toledo, Schwerzenbach, Switzerland) and an HC 113 electrode (Theta 90, Prague, Czech Republic). Three-point calibration was used at pH 4.0, 7.0, and 10.0. The honey samples were dissolved in distilled water without carbon dioxide and titrated with 0.1 mol/L sodium hydroxide solution (Penta, Prague, Czech Republic) to pH 8.3. Free acidity of honey was expressed in milliequivalents per kg of honey.

### 2.4. Bioactive Compounds

The total phenolic contents were analyzed spectrophotometrically with Folin–Ciocalteu solution and gallic acid as standard by the method according to Silici et al. [22]. Briefly, 1 g of honey was dissolved in 4 mL of methanol (Merck, Darmstadt, Germany) and then filtered through filter paper (Whatman, GE Health Care Limited, Buckinghamshire, United Kingdom). A total of40 µL of the filtrate was mixed with 2400 µL of distilled water, 200 µL of Folin–Ciocalteu solution (Penta, Prague, Czech Republic), and 600 µL of sodium carbonate solution (20%, *w*/*v*) (Penta, Prague, Czech Republic). After 2 h of incubation in the dark, the absorbance was measured at a wavelength of 765 nm against methanol as a blank using a Specord 200 Plus spectrometer (Analytic Jena AG, Jena, Germany). The total phenolic contents were evaluated using the gallic acid (Penta, Prague, Czech Republic) calibration curve in the concentration range of 0–900 mg/10 mL (R^2^ = 0.9990). After considering the weight of the sample, the total phenolic contents were expressed in mg of gallic acid equivalent (GAE) per 100 g of honey.

The total flavonoid contents were analyzed spectrophotometrically with quercetin as standard. Briefly, 1 g of honey was dissolved in 4 mL of methanol (Merck, Darmstadt, Germany) and then filtered through filter paper (Whatman, GE Health Care Limited, Buckinghamshire, United Kingdom). A total of 1500 µL of the filtrate was mixed with 60 µL of sodium nitrate (5%, *w*/*v*) (Penta, Prague, Czech Republic) and 60 µL of aluminum chloride (10%, *w*/*v*) (Penta, Prague, Czech Republic). The solution was incubated in the dark for 5 min. After 5 min, 400 µL of sodium hydroxide solution (1 mol/L) (Penta, Prague, Czech Republic) was added to the solution, and after 6 min of incubation, the absorbance was measured at a wavelength of 510 nm using a Specord 200 Plus spectrometer (Analytic Jena AG, Jena, Germany). The total flavonoid contents were evaluated using the quercetin (Merck, Darmstadt, Germany) calibration curve in the concentration range of 0–100 mg/L (R^2^ = 0.9950). After considering the weight of the sample, the total flavonoid contents were expressed in mg of quercetin equivalent (QE) per 100 g of honey.

Antioxidant activity was measured by detecting DPPH activity. The honey sample extracts were prepared by mixing 1.0 g of tempered, homogenized honey sample and 10.0 mL of distilled water (both tempered to 40 °C). Extraction was performed in an ultrasonic water bath (Sonorex Digitec, BANDELIN electronic GmbH & Co., KG, Berlin, Germany) for 30 min. Subsequently, the samples were filtered (injection filter 0.45 µm, Filtratech, Saint Jean de Braye, France) into clean Eppendorf tubes (approx. 2.0 mL). The honey extract was mixed with an ethanol solution containing DPPH radicals (0.0039 g of DPPH added to a volume of 100 mL with ethanol) (Sigma-Aldrich, Darmstadt, Germany). A total of 20 μL of the sample extracts and 180 μL of the DPPH solution were pipetted into the microtiter plate. A solution of 20 μL of distilled water and 180 μL of DPPH was used as the reference solution. The microtiter plate was incubated in the dark for 30 min. Subsequently, the absorbance at 517 nm was measured (Tecan Infinite M200, Schoeller Allibert, Prague, Czech Republic). Trolox solutions were used to prepare the calibration curve (Sigma-Aldrich, Darmstadt, Germany) on predetermined concentrations (0.0–1.6 mmol/L, R^2^ = 0.9810). Then, a calculation was performed according to the following equation:

DPPH Inhibition (%) = (A_initial_ − A_final_/A_initial_) × 100

The antioxidant capacity was determined using the FRAP assay. The extract of honey was prepared in the same way as for DPPH determination. Honey extract (75.0 µL) was mixed with FRAP reagent (1425.0 µL). The reaction mixture was then incubated in the dark at room temperature for 30 min. A total of 200 µL of the prepared mixtures was pipetted into the microtiter plate and the absorbance at 593 nm was measured. The FRAP reagent was prepared by mixing the acetic buffer (1.55 g of sodium octane—Penta Chemicals, Prague, Czech Republic + 8.0 mL of acetic acid—VWR, Prague, Czech Republic, add to 0.5 L with distilled water), TPTZ reagent (Sigma Aldrich, Darmstadt, Germany), and ferric chloride (VWR, Prague, Czech Republic). The calibration curve has been prepared in the same way as for DPPH (0.0–1.125 mmol/L, R^2^ = 0.9997). Subsequently, the calculation was carried out according to the following equation:

FRAP Antioxidant Capacity (%) = (A_sample_ − A_control_/A_max_) × 100

Color intensity was determined spectrophotometrically by the method according to Beretta et al. [23]. Briefly, 5.00 g of honey was weighed and dissolved in 5 mL of distilled water. The honey solution was filtered through a syringe filter 0.45 µm (Cronus Filter, Gloucester, UK) into a cuvette. Subsequently, absorbance was measured at wavelengths of 450 nm and 720 nm using a Specord 200 Plus spectrometer (Analytic Jena AG, Jena, Germany). Color intensity was calculated as the difference of absorbances at 450 nm and 720 nm multiplied by 1000 and expressed in mAU.

Color was determined spectrophotometrically using Honey Color Photometer Hanna HI96785 (Hanna Instruments, Jud. Salaj, Romania). The colorimeter was calibrated with glycerol standard reference (Hanna Instruments, Jud. Salaj, Romania). Color was expressed in units of mmPfund.

### 2.5. Statistical Analysis

Each result presented in this work represents an average value that was obtained from two independent analyses and the corresponding standard deviation. The obtained data were then statistically processed using Microsoft Excel 2016. To detect statistically significant differences, the normality test was used (Shapiro–Wilk Normality Test), and statistical significance was evaluated using the nonparametric Mann–Whitney U test. Statistical significances were assessed at the level of significance α = 0.05. Correlations were evaluated using the Pearson correlation. Statistical differences and correlations were evaluated in Unistat 6.5. A principal component analysis was performed in XLSTAT 2024.

## 3. Results and Discussion

### 3.1. Physicochemical Parameters

#### 3.1.1. Moisture Content

The moisture contents (g/100 g) of Kurdistan honeys varied from 13.3 ± 0.1 to 17.5 ± 0.1, with average moisture content 15.5 ± 1.2. All measured moisture contents of Kurdistan honeys are summarized in Table 2. The lowest moisture content was detected in sample KH11 from the foot of the mountains of the Akre region of Dohuk Governorate produced in 2022. Sample KH5 came from the same location, but from a different beekeeper and year of production 2018. On the contrary, the samples KH7 and KH9 with the two highest moisture contents came from the mountainous regions of Barbir and Atrush, regions of Dohuk Governorate, and were produced in 2018.

In agreement with our results, Azawy et al. [4] similarly determined the moisture content in the range of 15.6 to 16.6 g/100 g in three samples of honey from Halabja and Salamaniyah cities from Iraqi Kurdistan. Hussein et al. [5] detected a lower average moisture content (13.34 ± 1.39 g/100 g) in honeys from local apiaries in Iraqi Kurdistan. Khanbabaie et al. [7] also detected a lower average moisture content (13.79 g/100 g) in honeys from Kurdistan province of Iran. Furthermore, Emamifar and Hosseinpanahi [8] determined similar average moisture contents in Kurdistan honeys from Saghez (13.96 g/100 g) and Sanandaj (14.42 g/100 g) regions of Iranian Kurdistan. Comparable moisture contents (13.85 ± 3.75 g/100 g) were also detected by Parviz et al. [6] in the Kurdistan province of Iran. Adulkhaliq and Swaileh [24] detected higher average moisture content (16.53 ± 0.00 g/100 g) in honeys from West Bank, Palestine.

#### 3.1.2. Electrical Conductivity

The electrical conductivity of Kurdistan honeys ranged from 22.2 ± 0.6 mS/m to 49.8 ± 0.9 mS/m. Kurdistan honey samples with the lowest values of electrical conductivity came from wild bees from Barbir (KH7) and Gara (KH10) regions and from Shekhka region (KH12). The average electrical conductivity of Kurdistan honeys was 32.3 ± 7.4 mS/m and according to European Union law, all honeys can be classified as floral honeys. All measured electrical conductivities of Kurdistan honeys are summarized in Table 2.

Electrical conductivities similar to the values detected by us were measured in honeys from Iraqi Kurdistan by Azawy et al. [4]. In contrast, Hussein et al. [5] detected a higher average value of electrical conductivity (43.8 ± 4.7 mS/m) in honeys from Iraqi Kurdistan. Similar average values of electrical conductivity were determined by Emamifar and Hosseinpanahi [8] in Saghez honeys (36 mS/m) and Sanandaj honeys (22 mS/m) from the Kurdistan province of Iran. The lower electrical conductivity values determined in honey from wild bees probably reflected specific and different nectar preferences of these bees. The samples of Kurdistan honeys came from places with diverse flora, including various herbs and trees, as specified in Table S1. Considering the diverse plant availability and sampling locations, it was interesting to observe relatively low variability in electrical conductivity and the absence of honeydew honey samples. The reason for the absence of a sample of honeydew honey may be unfavorable climatic conditions or a low incidence of honeydew producers.

#### 3.1.3. Diastase Activity

The diastase activity was a floating parameter in Kurdistan honeys, ranging from 4.7 ± 0.2 DN to 23.4 ± 1.5 DN, as indicated in Table 2. The average value of diastase activity was 14.1 ± 5.6 DN. The lowest diastase activity was detected in sample KH2, and this sample came from a home garden in the city of Dohuk. The sample with the highest detected diastase activity, KH6, came from a mountainous area of Brifka region with a predominance of trees and flowering wild weeds. The detected different values pointed to the influence of the city/mountain location and available flora on diastase activity.

Compared to us, Hussein et al. [5] detected about twice the average diastase activity (32.01 ± 3.87 DN) in honeys from local apiaries in Iraqi Kurdistan. Emamifar and Hosseinpanahi [8] reported similar average diastase activities in Saghez honeys (11.3 DN) and Sanandaj honeys (15.26 DN) from the Kurdistan province of Iran. Lower diastase activities also under the limit (not less than 8 DN) from 1.00 DN to 15.95 DN were detected by Afshari et al. [25] in honey samples from the Khorasan province of Iran. In contrast, Parviz et al. [6] detected in honeys from the Kurdistan province of Iran higher average diastase activity (29.7 ± 0.92 DN). Diastase activity is a very variable parameter, which was also pointed out by the differences between the values detected, and the values found by other authors. Diastase is an enzyme, specifically α-amylase, characterized as an unstable parameter of honey, and this could be one of the reasons for its variable value.

#### 3.1.4. Hydroxymethylfurfural Content

The HMF contents (mg/kg) of honeys from Kurdistan beekeepers ranged from 5.0 ± 0.1 to 65.9 ± 0.5, with an average HMF concentration of 16.3 ± 14.9. The highest concentration of HMF was detected in sample KH13 from Dohuk city and came from the production of 2022. This was an outlier, as indicated by the other detected HMF concentrations (from 5.0 mg/kg to 32.0 mg/kg) and the median, as indicated in Table 2. The reason for the high determined concentration of HMF in sample KH13 could be climatic conditions, inappropriate heating, or inappropriate storage of honey by the beekeeper.

A very similar average concentration of HMF (17.2 ± 6.4 mg/kg) was detected by Hussein et al. [5] in honeys from local apiaries in Iraqi Kurdistan. Emamifar and Hosseinpanahi [8] determined lower average HMF contents in Saghez honeys (9.26 mg/kg) and Sanandaj honeys (5.63 mg/kg) from the Kurdistan province of Iran. In contrast, Parviz et al. [6] detected in honeys from the Kurdistan province of Iran several times lower average HMF content (0.92 ± 0.38 mg/kg). Like us, Afshari et al. [25] and Abdulkhaliq and Swaileh [24] detected in honey samples from Khorasan province of Iran and honeys from Palestine higher HMF content, even above 30 mg/kg. The concentration of HMF is a very variable parameter of honey. Variation in our results in comparison with other authors indicated variability of this parameter depending on several factors, like climatic conditions, and impact of honey processing or handling on honey quality.

#### 3.1.5. Free Acidity and pH

The free acidity (meq/kg) of Kurdistan honeys ranged from 21.0 ± 0.7 to 49.5 ± 1.2 with average value 31.1 ± 7.2. None of the tested samples showed signs of fermentation, the presence of bubbles, or a change in smell. The highest acidity value was detected in the KH5 sample, as indicated in Table 2. This sample came from the 2018 production of the foot of a mountain region of Akre. Despite the high detected acidity, the KH5 sample did not show signs of fermentation and ongoing microbial activity, and therefore, the high acidity can be attributed to the specificity of plant origin.

Very similar free acidities were detected by Hussein et al. [5] and Azawy et al. [4] in honeys from Iraqi Kurdistan. Parviz et al. [6] detected in honeys from the Kurdistan province of Iran similar acidity (23.05 ± 2.29 meq/kg). Similar results of acidity have been detected in other studies. Emamifar and Hosseinpanahi [8] determined acidity in Saghez honeys (25.22 meq/kg) and Sanandaj honeys (29.35 meq/kg) and Khanbabaie et al. [7] determined acidity (21.39 meq/kg) in honeys from the Kurdistan province of Iran.

The pH values of Kurdistan honeys were very close and varied from 3.5 ± 0.0 to 3.9 ± 0.0. The average pH value of Kurdistan honeys was 3.7 ± 0.1. Very close to our detected pH values were also pH values (3.86 and 3.87) detected by Khanbabaie et al. [7] and Emamifar and Hosseinpanahi [8] in honeys from the Kurdistan province of Iran. In contrast, Hussein et al. [5] and Azawy et al. [4] detected in honeys from Iraqi Kurdistan higher pH values, from 4.10 to 4.81 and 4.08 ± 0.15 on average.

#### 3.1.6. Saccharide Content

Kurdistan honey samples from the 2022 production (KH11-KH16) were analyzed for saccharide content. The presence of fructose and glucose was detected and quantified in all analyzed samples. Sucrose was below the limit of detection for all samples (<0.3 g/100 g). The main carbohydrate in the analyzed Kurdistan honeys was fructose with an average value of 39.6 ± 2.1 g/100 g. For comparison, the average glucose content was lower, at 31.5 ± 3.3 g/100 g, which was also reflected in the fructose-to-glucose ratios, which were from 1.0 to 1.5. The predominance of fructose content over glucose is manifested in the liquid state of honey, which was also observed in the samples analyzed by us. The highest fructose content was detected in sample KH15 originating from the Matin mountain range of Bamerne region. The sum of fructose and glucose contents ranged from 66.3 to 76.4 g/100 g and was typical for floral honeys (higher than 60 g/100 g), which corresponded to the measured electrical conductivities. Individual saccharide contents in Kurdistan honeys are presented in Table 3.

The determined average content of the sum of fructose and glucose (71.1 ± 3.3 g/100 g) corresponded to the average value of reducing sugars determined by Azawy et al. [4] (77.0 ± 1.3 g/100 g) in honeys from Iraqi Kurdistan and by Khanbabaie et al. [7] (77.67 g/100 g) in honeys from the Kurdistan province of Iran. In contrast, the same authors determined a higher average sucrose content 2.0 ± 0.76 and 2.22 g/100 g. Also, Emamifar and Hosseinpanahi [8] determined similar average reducing sugar contents in Kurdistan honeys from Saghez (74.9 g/100 g) and Sanandaj (79.09 g/100 g) regions. The same authors also detected similar fructose-to-glucose ratios to us (1.26 and 1.16). Emamifar and Hosseinpanahi [8] also determined higher mean sucrose contents, 1.86 g/100 g and 1.1 g/100 g, compared to the values detected by us. Comparable average sucrose content (0.13 ± 0.04 g/100 g) was detected by Parviz et al. [6] in honeys from Kurdistan province of Iran. The cause of different values of sucrose content can be specific plant sources or feeding bees with carbohydrate syrups.

### 3.2. Bioactive Compounds

#### 3.2.1. Total Phenolic Content

The results showed that the determined total phenolic contents (mg GAE/100 g) in Kurdistan honeys varied greatly, from 30.8 ± 3.9 to 79.6 ± 6.6, as presented in Table 4. The average value of total phenolic contents was 55.1 ± 14.2 mg GAE/100 g. The highest total phenolic content was detected in sample KH11 from the foot of the mountains from Akre region of Dohuk Governorate produced in 2022. For this sample, we also detected the second highest electrical conductivity. In contrast, the lowest total phenolic content was detected in honey sample KH10 from wild bees from Gara mountain range. The electrical conductivity of the KH10 sample was the third lowest.

The values of the total phenolic content determined by us were also higher compared to the results of Emamifar and Hosseinpanahi [8] in Saghez honeys (48.36 mg gallic acid/kg) and Sanandaj honeys (34.22 mg gallic acid/kg) from the Kurdistan province of Iran. The total phenolic content (mg GAE/100 g) of Kurdistan honeys was also higher compared to the total phenolic content of European unifloral honeys presented by Tomczyk et al. [26] in black locust (20 ± 5), rape (21 ± 4), and lime (35 ± 1) honeys directly from beekeepers. On the other hand, Azawy et al. [4] determined a several times higher total phenolic content in honeys from Iraqi Kurdistan, with an average value of 101 ± 8.04 mg GAE/100 g.

#### 3.2.2. Color and Color Intensity

Like total phenolic content, the color (mm Pfund) of Kurdistan honeys was also a very variable parameter, ranging from 10.0 ± 0.5 to 132.0 ± 1.4 with an average value of 55.2 ± 33.2. The lightest sample with the lowest detected color value was honey KH8 from Bestana of Erbil Governorate with the main source of nectar originating from Eucalyptus trees. The second lowest total phenolic content was also detected in this sample. The darkest honey with the highest detected color value was sample KH11, which also had the highest total phenolic content.

According to USDA color classification [27], the color of the Kurdistan honey samples varied from extra white to dark, as presented in Table 4. Most samples (38%) were classified as light amber, and the second most represented group was white honeys (31%). There was only one sample each in the extra white, dark, and amber categories.

The color intensity (mAU) of the Kurdistan honeys ranged from 154.0 ± 17.0 to 687.9 ± 20.6, with an average value of 338.5 ± 153.8. The lowest color intensity was detected in the white honey sample KH6 from the mountainous region of Brifka. This area is dominated by oaks, junipers, and *Crataegus* and flowers of various wild weeds. The highest color intensity was detected in dark honey sample KH11, which also had the highest color value, the highest amount of total phenolic substances, and the lowest moisture content. The KH11 sample came from the Akre region, where citrus, almonds, wild walnuts, oaks, daffodils, anemone, *Rosa davidii*, thyme, coriander, and Tagetes dominate. Observed variations in determined values of color intensity indicate the influence of several pre-extraction factors such as the presence of various plant pigments, pollen grains, and phenolic substances on the color intensity of honey.

#### 3.2.3. Antioxidant Activity and Total Flavonoid Content

Kurdistan honey samples from the 2022 production (KH11-KH16) were analyzed for free radical scavenging activity (DPPH), ferric reducing/antioxidant power (FRAP), and total flavonoid content. DPPH inhibition (%) in the analyzed Kurdistan honeys varied from 3.3 ± 1.4 to 35.8 ± 2.0, with an average value of inhibition of 27.0 ± 11.9. The highest value of antioxidant activity expressed by the highest % value of inhibition was detected in sample KH12 from the Shekhka region.

Total flavonoid content (mg QE/100 g) in the analyzed Kurdistan honeys varied from 23.0 ± 0.1 to 43.4 ± 0.5, with an average value of 27.9 ± 7.8. The percentage of FRAP antioxidant capacity (%) ranged from 17.7 ± 0.1 to 47.0 ± 0.1, with an average value of antioxidant capacity of 25.2 ± 11.0. The antioxidant capacities of the analyzed samples of Kurdistan honeys were very close, as indicated by the values shown in Table 5, with one outlier value for sample KH11. The highest antioxidant capacity (FRAP) and total flavonoid content were detected in the KH11 sample. The same sample also had the highest total phenolic content and was the darkest sample with the highest color and color intensity values. On the contrary, the lowest antioxidant capacity and total flavonoid content were measured in sample KH12, and in this sample was also detected the lowest total phenolic content. These findings indicated that the antioxidant capacity and total flavonoid content of the Kurdistan honey samples correlated with the total content of phenolic substances. The percentage of DPPH antioxidant activity of honeys from Kurdistan provinces of Iran was analyzed by Emamifar and Hosseinpanahi [8]. The same authors determined higher average values of antioxidant activity in honeys from cities Saghez (58.69%) and Sanandaj (41.23%) compared to the average value detected by us. Higher values of antioxidant activity (51.6%) were also detected in honeys from Iraqi Kurdistan by Azawy et al. [4]. The limiting factor for an adequate assessment of the antioxidant properties of Kurdistan honeys was the low number of samples investigated.

#### 3.2.4. Correlations

Pearson correlation coefficients between parameters were determined for Kurdistan honeys. All correlation coefficients are presented in Table 6. No very high correlation (*r* > 0.90) was detected. A high positive correlation was identified between the electrical conductivity and the color of honey (*r* = 0.713). Electrical conductivity was also correlated with the total phenolic content; a moderate positive correlation was detected between these parameters (*r* = 0.615). The color intensity of honeys depended on the total phenolic content; a moderate positive correlation was detected between these parameters (*r* = 0.659). Tomczyk et al. [26] detected a very similar correlation between color intensity and total phenolic content (*r* = 0.703). Moderate positive correlation was also detected between color and HMF content (*r* = 0.536). HMF is a colorless compound, an intermediate product of the Maillard reaction, from which the dark-colored final products are formed.

### 3.3. Comparison of Kurdistan Honeys with European Union Law

All Kurdistan honeys have been compared with the requirements of European Union law relating to honey, specifically with the requirements resulting from Council Directive of the EU 110/2001 [20] related to honey. This comparison is summarized in Table 7.

All analyzed samples of Kurdistan honeys met the limit related to moisture content, electrical conductivity, sum of fructose and glucose, sucrose content, and free acidity. Only one honey sample, KH13, had an HMF concentration higher than the limit of 40 mg/kg, specifically 69.5 ± 0.5 mg/kg. According to the requirements of Council Directive of the EU 110/2001 [20], honeys from regions with tropical climate have a set limit of HMF max. 80 mg/kg. Geographically, Iraqi Kurdistan belongs to the subtropical region, and therefore, it is not possible to apply this exception. Diastase activity lower than 8 DN was detected in two honey samples (KH2 and KH8). For diastase activity, Council Directive of the EU 110/2001 [20] established an exception from diastase activity, for example, for citrus honeys, it allows a minimum diastase activity of 3 DN and an HMF content max. 15 mg/kg. Application of the exception would be possible after determining the botanical origin of the honey.

### 3.4. Comparison of Kurdistan Honeys with Czech and Slovak Honeys

#### 3.4.1. Statistical Significance

The properties of Kurdistan honeys were compared with the properties of honeys originating from Central Europe, specifically with honeys originating from the Czech and Slovak Republics. No statistically significant difference (*p* > 0.05) was detected in the value of electrical conductivity and color between honeys from Kurdistan and honeys from the Czech and Slovak republic. Similarly, a statistically insignificant difference (*p* > 0.05) was also detected in the content of fructose, glucose, sucrose, free acidity, pH, DPPH, and FRAP between Kurdistan honeys and honeys from the Czech Republic. A statistically significant difference (*p* < 0.05) between Kurdistan and Czech honeys was detected in moisture content, diastase activity, HMF content, total phenolic content, and total flavonoid content. Specifically, compared to Czech honeys, Kurdistan honeys had a lower moisture content, lower diastase activity, lower total flavonoid content, and higher HMF content and a higher total phenolic content. Between Kurdistan and Slovak honeys was detected a statistically significant difference (*p* < 0.05) in HMF content, total phenolic content, and color intensity. Despite the warmer climate of Iraqi Kurdistan, diastase activities of Kurdistan honeys were very similar to diastase activities of honeys from the Slovak Republic (*p* > 0.05). All detected statistical significances between Kurdistan honeys and honeys from the Czech and Slovak Republics are presented in Table 8.

Based on the measured results and statistical significances, it can be concluded that the properties of Kurdistan honeys were very close to the properties of Czech and Slovak honeys, even though the honeys came from different climatic regions and places with different flora. The different climatic conditions, including the subtropical climate of Kurdistan honeys, could be the main reason for the higher detected concentration of HMF and lower moisture content compared to honeys from a continental climate. In addition, Kurdistan honeys had a statistically significantly (*p* < 0.05) higher total phenolic content and very similar DPPH and FRAP values compared to European honeys. The total phenolic content is important from the point of view of the nutritional value of honey and health benefits, because the total phenolic content indicates the content of phenolic substances, which are associated with antiviral, antimicrobial, antioxidant, anti-inflammatory, antiatherogenic, and anticancer properties of honey [13].

The chemical composition of honey also affects its rheological properties. According to Saxena et al. [28], the viscosity of honey and its consistency properties are related to moisture content and water activity. The same authors detected a very high negative correlation (*r* = −0.9308) between water activity and viscosity. From the point of view of consistency properties, differences were observed between honeys from Iraqi Kurdistan compared to honeys from the Czech and Slovak Republics. Honeys from Iraqi Kurdistan were consistently different, denser than European honeys, which was probably a consequence of the lower moisture content.

#### 3.4.2. Principal Component Analysis

**Figure 1:**
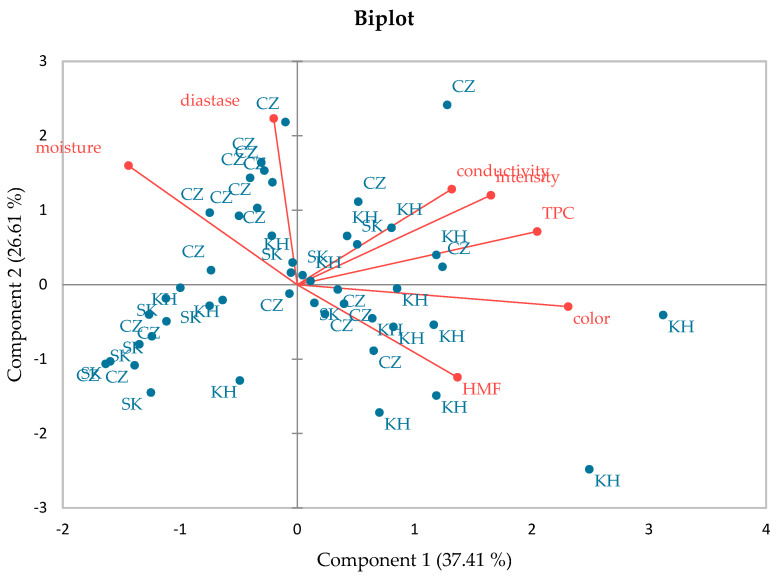
Distribution of honey samples according to geographical origin by applying the principal component analysis, based on the analysis of moisture content, electrical conductivity, hydroxymethylfurfural (HMF) content, diastase activity, color, color intensity, and total phenolic content (TPC), and the selection of two main components. The sample code indicates the geographical origin (CZ: Czech honeys, SK: Slovak honeys, KH: Iraqi Kurdistan honeys).

A principal component analysis was performed to assess the relationship between the geographical origin of honey and physicochemical parameters. For the principal component analysis, were used parameters that were determined in all 48 honey samples (moisture content, electrical conductivity, HMF content, diastase activity, color, color intensity, and total phenolic content). Based on the evaluation of the calculated eigenvalues (for component 1: 2.619 and for component 2: 1.863), two components were selected. The parameters with the highest weights in the first component were color (0.545), total phenolic content (0.483), and color intensity (0.389). In the second component, diastase activity (0.625), moisture content (0.447), and electrical conductivity (0.359) had the highest weight. The graphic presentation of the first two components is presented in Figure 1.

#### 3.4.3. Limitation of Study

The main limiting factor of this study was the number of samples from Iraqi Kurdistan which were collected and analyzed. The number of samples taken in a given year was based on the conditions that allowed sampling, such as a sufficient amount of honey produced by the beekeeper, suitable climatic conditions, and also the possibility of obtaining honey from wild bees. Therefore, an opportunity for improvement is to carry out further sampling and coverage of the entire extent of Iraqi Kurdistan. Another limiting factor is the scope of the performed analyses. The scope of analyses for honey samples taken in 2018 and 2022 was not identical due to the instrumental and material possibilities in the given year and the amount of honey taken from Kurdish beekeepers. The mentioned limitations bring an opportunity for further analyses and study.

## 4. Conclusions

A whole range of physicochemical and bioactive parameters related to the quality and nutritional properties of honey was determined. The obtained results supplemented the insufficient data regarding the properties of honeys originating from Iraqi Kurdistan, specifically from Dohuk and Erbil Governorates. It was detected that the honeys produced in Iraqi Kurdistan met the strict quality criteria set by European Union law, which is important information for local beekeepers and experts in relation to honey production and an opportunity to set local limits of honey quality. Despite the different climatic and topographical conditions, the available flora, and the different species of bees, the properties of Iraqi Kurdistan honeys were similar to the properties of honeys from the Czech and Slovak Republics. This fact was pointed out by statistically insignificant differences (*p* > 0.05) in the content of fructose, glucose, sucrose, free acidity, pH, DPPH, FRAP, electrical conductivity, color, and color intensity between Kurdistan honeys and honeys from the Czech Republic. In terms of biological effects, although Kurdistan honeys had a lower total flavonoid content compared to Czech honeys, on the contrary, they had a statistically significantly (*p* < 0.05) higher total phenolic content. From the point of view of the principal component analysis, the color, total phenolic content, and color intensity were the main parameters affecting the characteristics of the analyzed honeys. This work opened the opportunity for further analysis of samples originating from the Iraqi Kurdistan region, in terms of a deeper assessment of the properties of honeys and the number of analyzed samples.

## Figures and Tables

**Table 1:** Additional information on honey samples from Iraqi Kurdistan.

Sample	Area	Location	Origin	Production
KH1	Dohuk city	36°50′17.2″ N 42°59′12.5″ E	beekeeping	9-10/2018
KH2	Dohuk city	36°50′28.3″ N 42°59′14.5″ E	beekeeping	9-10/2018
KH3	Sheikh Hasn	36°47′30.2″ N 43°12′30.4″ E	beekeeping	9-10/2018
KH4	Sitke	36°45′28.9″ N 43°03′44.1″ E	beekeeping	9-10/2018
KH5	Akre	36°44′02.8″ N 43°52′43.5″ E	beekeeping	9-10/2018
KH6	Brifka	36°48′47.8″ N 43°13′21.0″ E	beekeeping	9-10/2018
KH7	Barbir	36°47′23.9″ N 43°17′44.3″ E	wild	9-10/2018
KH8	Bestana	36°02′44.0″ N 44°11′57.6″ E	beekeeping	9-10/2018
KH9	Atrush	36°50′12.8″ N 43°20′04.0″ E	beekeeping	9-10/2018
KH10	Gara	37°00′39.6″ N 43°22′00.9″ E	wild	9-10/2018
KH11	Akre	36°44′02.8″ N 43°52′43.5″ E	beekeeping	9-10/2022
KH12	Shekhka	36°46′04.8″ N 43°26′57.6″ E	beekeeping	9-10/2022
KH13	Dohuk city	36°50′28.3″ N 42°59′14.5″ E	beekeeping	9-10/2022
KH14	Benarinke	36°53′08.4″ N 43°14′44.1″ E	beekeeping	9-10/2022
KH15	Bamerne	37°06′47.8″ N 43°16′14.0″ E	beekeeping	9-10/2022
KH16	Bade	36°54′21.6″ N 43°05′40.5″ E	beekeeping	9-10/2022

**Table 2:** Physicochemical parameters of the analyzed Kurdistan honeys.

Samples	Moisture (g/100 g)	Conductivity (mS/m)	Diastase (DN)	HMF (mg/kg)	Free Acidity (meq/kg)	pH
KH1	15.5 ± 0.4	31.4 ± 2.1	19.6 ± 0.0	8.4 ± 0.1	36.5 ± 1.2	3.5 ± 0.0
KH2	16.9 ± 0.1	32.3 ± 1.2	4.7 ± 0.2	15.2 ± 0.4	37.0 ± 1.0	3.5 ± 0.0
KH3	16.1 ± 0.0	33.9 ± 1.7	13.9 ± 0.7	13.4 ± 2.0	28.0 ± 1.3	3.7 ± 0.0
KH4	15.3 ± 0.2	34.4 ± 3.4	19.9 ± 0.5	15.2 ± 1.7	33.0 ± 0.5	3.9 ± 0.0
KH5	15.8 ± 0.0	49.8 ± 0.9	21.7 ± 1.3	11.0 ± 1.0	49.5 ± 1.2	3.9 ± 0.0
KH6	16.2 ± 0.0	29.8 ± 1.4	23.4 ± 1.5	11.5 ± 0.3	41.5 ± 0.8	3.7 ± 0.0
KH7	17.4 ± 0.1	22.2 ± 0.6	12.7 ± 0.4	8.4 ± 1.2	30.0 ± 1.0	3.5 ± 0.0
KH8	15.0 ± 0.1	27.3 ± 3.5	6.0 ± 0.4	11.0 ± 1.5	21.0 ± 0.7	3.6 ± 0.0
KH9	17.5 ± 0.1	31.7 ± 0.0	11.7 ± 0.2	5.0 ± 0.4	32.0 ± 0.2	3.9 ± 0.0
KH10	16.6 ± 0.2	24.2 ± 0.5	19.1 ± 0.3	5.0 ± 0.1	26.0 ± 1.8	3.7 ± 0.0
KH11	13.3 ± 0.1	47.1 ± 0.2	8.8 ± 0.3	10.8 ± 1.0	30.0 ± 0.0	3.7 ± 0.0
KH12	14.2 ± 0.0	23.8 ± 0.0	11.0 ± 0.1	25.6 ± 0.1	24.5 ± 0.0	3.6 ± 0.0
KH13	14.0 ± 0.0	32.0 ± 0.2	9.8 ± 0.6	65.9 ± 0.5	29.3 ± 0.4	3.5 ± 0.0
KH14	14.6 ± 0.0	29.7 ± 0.2	10.8 ± 0.4	32.0 ± 0.0	27.3 ± 0.4	3.6 ± 0.0
KH15	14.6 ± 0.1	33.1 ± 0.3	15.1 ± 0.7	11.1 ± 0.4	25.0 ± 0.0	3.7 ± 0.0
KH16	15.0 ± 0.0	33.5 ± 0.1	16.8 ± 0.2	12.0 ± 0.4	26.8 ± 0.0	3.7 ± 0.0
Min.	13.3	22.2	4.7	5.0	21.0	3.5
Max.	17.5	49.8	23.4	65.9	49.5	3.9
Median	15.4	31.9	13.3	11.3	29.6	3.7

Data are presented as average ± standard deviation. HMF—hydroxymethylfurfural.

**Table 3:** Saccharide content of analyzed Kurdistan honeys from 2022.

Samples	Fructose (g/100 g)	Glucose (g/100 g)	Sucrose (g/100 g)	Sum F + G (g/100 g)	Ratio F/G
KH11	38.7 ± 6.3	37.6 ± 5.5	<0.3	76.4 ± 1.0	1.0
KH12	37.6 ± 5.1	28.8. ± 4.0	<0.3	66.3 ± 1.3	1.3
KH13	38.8 ± 0.9	32.1 ± 1.2	<0.3	70.9 ± 1.2	1.2
KH14	39.5 ± 0.3	30.8 ± 0.3	<0.3	70.3 ± 1.3	1.3
KH15	43.8 ± 1.3	28.7 ± 1.1	<0.3	72.5 ± 1.5	1.5
KH16	39.1 ± 1.8	30.9 ± 3.9	<0.3	70.0 ± 1.3	1.3
Min.	37.6	28.7	-	66.3	1.0
Max.	43.8	37.6	-	76.4	1.5
Median	38.9	30.8	-	70.6	1.3

Data are presented as average ± standard deviation. F—fructose, G—glucose.

**Table 4:** Total phenolic content and color of the analyzed Kurdistan honeys.

Samples	TPC (mg GAE/100 g)	Color Intensity (mAU)	Color (mm Pfund)	Color Grade
KH1	57.0 ± 8.6	245.0 ± 2.8	30.0 ± 3.7	white
KH2	73.6 ± 0.7	321.0 ± 24.0	79.4 ± 5.3	light amber
KH3	67.2 ± 4.3	354.5 ± 29.0	55.8 ± 5.0	light amber
KH4	64.3 ± 9.1	598.5 ± 46.0	49.7 ± 3.7	extra light amber
KH5	59.5 ± 7.1	435.0 ± 52.3	43.0 ± 0.0	extra light amber
KH6	61.3 ± 8.2	154.0 ± 17.0	27.4 ± 1.1	white
KH7	48.7 ± 8.1	183.0 ± 11.3	22.8 ± 0.8	white
KH8	34.8 ± 4.6	228.5 ± 6.4	10.0 ± 0.5	extra white
KH9	71.4 ± 8.7	513.0 ± 36.8	31.9 ± 0.0	white
KH10	30.8 ± 3.9	195.0 ± 7.1	17.4 ± 0.5	white
KH11	79.6 ± 6.6	687.9 ± 20.6	132.0 ± 1.4	dark
KH12	38.3 ± 7.8	218.8 ± 3.3	70.5 ± 0.7	light amber
KH13	45.8 ± 9.7	404.6 ± 2.4	106.0 ± 1.4	amber
KH14	47.6 ± 6.1	302.7 ± 1.6	74.0 ± 0.0	light amber
KH15	54.5 ± 6.4	291.9 ± 13.4	65.0 ± 0.0	light amber
KH16	47.3 ± 4.6	282.8 ± 4.9	68.0 ± 0.0	light amber
Min.	30.8	154.0	10.0	extra white
Max.	79.6	687.9	132.0	dark
Median	55.7	297.3	52.8	light amber

Data are presented as average ± standard deviation. TPC—total phenolic content.

**Table 5:** Antioxidant properties and total flavonoid content of analyzed Kurdistan honeys from 2022.

Samples	DPPH (%)	FRAP (%)	TFC (mg QE/100 g)
KH11	3.3 ± 1.4	47.0 ± 0.1	43.4 ± 0.5
KH12	35.8 ± 2.0	17.7 ± 0.1	23.0 ± 0.1
KH13	31.1 ± 0.4	19.3 ± 0.2	26.4 ± 0.1
KH14	27.4 ± 0.9	20.3 ± 0.0	27.3 ± 0.0
KH15	31.0 ± 2.2	25.6 ± 0.3	24.6 ± 0.0
KH16	33.3 ± 1.0	21.0 ± 0.3	23.0 ± 0.1
Min.	3.3	17.7	23.0
Max.	35.8	47.0	43.4
Median	31.1	20.7	25.5

Data are presented as average ± standard deviation. TFC—total flavonoid content.

**Table 6:** Pearson correlation coefficients analyzed in Kurdistan honeys from 2018 and 2022.

*n* = 16	Moisture	Conductivity	HMF	Diastase	Color	Color int.	TPC
Moisture	1						
Conductivity	−0.316	1					
HMF	−0.496	−0.060	1				
Diastase	0.185	0.153	−0.304	1			
Color	−0.672	0.465	0.536	−0.416	1		
Color int.	−0.290	0.713	0.071	−0.133	0.552	1	
TPC	0.129	0.615	−0.225	−0.037	0.382	0.659	1

HMF—hydroxymethylfurfural, TPC—total phenolic content.

**Table 7:** Comparison of Kurdistan honeys with Council Directive of the EU related to honey.

Parameters	Determined Values	Limit EU 110/2001	Samples Exceeding Limit
Moisture (g/100 g)	max. 17.5	max. 20	0
Conductivity (mS/m)	max. 49.8	max. 80	0
HMF (mg/kg)	max. 69.5	max. 40	1
Diastase (DN)	min. 4.7	min. 8	2
Fructose + glucose (g/100 g)	min. 66.3	min. 60	0
Sucrose (g/100 g)	max. 0.3	max. 5	0
Free acidity (meq/kg)	max. 49.5	max. 50	0

HMF—hydroxymethylfurfural, EU—European Union.

**Table 8:** Comparison of Kurdistan honeys with Czech and Slovak honeys.

Year of Production 2018 and 2022	Kurdistan (*n* = 16)	Czech (*n* = 22)	Slovak (*n* = 10)
Moisture (g/100 g)	15.5 ± 1.2 ^a^	17.0 ± 1.1 ^b^	16.4 ± 0.9 ^a^
Conductivity (mS/m)	32.3 ± 7.4 ^a^	38.0 ± 18.2 ^a^	33.7 ± 19.1 ^a^
Diastase (DN)	14.1 ± 5.6 ^a^	21.0 ± 7.8 ^b^	14.4 ± 4.2 ^a^
HMF (mg/kg)	16.3 ± 14.9 ^a^	6.8 ± 3.8 ^b^	3.8 ± 2.1 ^c^
TPC (mg GAE/100 g)	55.1 ± 14.2 ^a^	41.8 ± 11.1 ^b^	34.0 ± 11.4 ^b^
Color (mm Pfund)	55.2 ± 33.2 ^a^	45.0 ± 22.9 ^a^	39.4 ± 21.3 ^a^
Color int. (mAU)	338.5 ± 153.8 ^a^	249.1 ± 193.9 ^a^	71.5 ± 44.5 ^b^
**Year of production 2022**	**Kurdistan** **(*n* = 6)**	**Czech** **(*n* = 12)**	-
Fructose (g/100 g)	39.6 ± 2.1 ^a^	38.7 ± 2.4 ^a^	-
Glucose (g/100 g)	31.5 ± 3.3 ^a^	32.2 ± 2.8 ^a^	-
Sucrose (g/100 g)	> 0.3 ^a^	> 0.3 ^a^	-
Free acidity (meq/kg)	27.2 ± 2.2 ^a^	26.0 ± 7.4 ^a^	-
pH	3.6 ± 0.1 ^a^	3.9 ± 0.2 ^a^	-
DPPH (%)	27.0 ± 11.9 ^a^	28.5 ± 3.4 ^a^	-
FRAP (%)	25.2 ± 11.0 ^a^	21.0 ± 5.1 ^a^	-
TFC (mg QE/100 g)	27.9 ± 7.8 ^a^	36.1 ± 16.8 ^b^	-

^abc^—different letters indicate a statistically significant difference (*p* < 0.05), HMF—hydroxymethylfurfural, TPC—total phenolic content, TFC—total flavonoid content. Data are presented as average ± standard deviation.

## Data Availability

The original contributions presented in the study are included in the article, further inquiries can be directed to the corresponding author.

## Supplementary Materials

The following supporting information can be downloaded at: https://www.mdpi.com/article/10.3390/foods13142228/s1, Table S1: Additional information on honey samples from Iraqi Kurdistan.; Table S2: Additional information on honey samples from Czech and Slovak beekeepers.
