# Supplementary material for: Quality Characterization of Honeys from Iraqi Kurdistan and Comparison with Central European Honeys
Source: Foods. 2024 Jul 16;13(14):2228. doi: 10.3390/foods13142228 (PMC11275447; doi:10.3390/foods13142228)
Supplement: Supplementary file 1 [file foods-13-02228-s001.zip › foods-3065329-supplementary.pdf]

Table S1. Additional information on honey samples from Iraqi Kurdistan.

Sample	Character of the location	Important flora	Position
KH1	The foot of a mountain overlooking the city of Dohuk.	Figs, almonds, grapes, olives, Pirus, Syriaca, and wild plum. As for the weeds of this mountain, such as *Malva parviflora* and Chamomile.	https://maps.app.goo.gl/Y4D44DvDYZHGkZfr7
KH2	Inside the home garden in the city of Dohuk.	Peach, apricot, grape, Loquat and citrus fruits such as oranges and lemons, as well as vegetables such as eggplant, pepper, okra, and celery.	https://maps.app.goo.gl/KLa3MpUw5SGiHBH49
KH3	The area is mountainous.	Oak trees, junipers and *Crataegus*, flowers of various wild weeds.	https://maps.app.goo.gl/ZowJhKnaNtN2aoGB9
KH4	Southeast of Dohuk, an undulating plain area, considered one of the agricultural areas.	Its most important crops are the legumes chickpeas, lentils, beans, coriander, and sesame, in addition to wheat, barley, corn, sunflowers, and cotton, in addition to wild herbs such as Poppy, anemones, wild daffodils, wild berries, wolfberries, and willows, in addition to trees Plum, olive, citrus, eucalyptus.	https://maps.app.goo.gl/cr18v3k5YbSMNZMT9
KH5	The foot of a mountain overlooking a plain.	Wheat and barley and oil plants such as sunflowers and cotton, in addition to citrus and olive trees, as well as trees on the surface of the mountain such as Thuja, almonds, wild walnuts, oaks and Crataegus. Among the most important flowers are daffodils, anemone, *Rosa davidii*, thyme, Coriander and Tagetes.	https://maps.app.goo.gl/ji8sPh6uEQyvAwhq9
KH6	The area is mountainous.	Oak trees, junipers and *Crataegus*, flowers of various wild weeds. Feeding: the breeder feeds the bees sugar only when it is needed and there is no natural source, it is for a short time in the winter.	https://maps.app.goo.gl/YTfzet6GpHJdXLSs8
KH7	The area is mountainous.	Oak trees, junipers, almond and *Crataegus* trees, flowers of various wild weeds.	https://maps.app.goo.gl/4EbcvUiHbEeTmvjs9
KH8	The region has a flat geographical nature.	The breeder does not feed sugar to the bees, i.e. they depend on natural nutrition, the main source of feed is the Eucalyptus tree.	https://maps.app.goo.gl/E31fgHiaGLKSRxoK7
KH9	The top of a high mountain overlooking a plain.	Pine, juniper, oak, wild walnut, and wild plum. Peas, chickpeas, and lentils are grown there, in addition to wheat and barley. Weeds (wild plants) such as *Malva parviflora*, Chamomile, and catnip.	https://maps.app.goo.gl/UDqWrX5YGQ3SK7ie9
KH10	The Gara mountain range, famous in Iraq for its summer resorts.	Oak, thuja, hawthorn, *Pistacia terebinthus*, juniper, pine and wild almond, as for its wildflowers such as the *Fritillaria imperialis*, narcissus, Chamomile and anemone.	https://maps.app.goo.gl/1W6koVC7gnZDcWfo8

Table S1. Additional information on honey samples from Iraqi Kurdistan.

Sample	Character of the location	Important flora	Position
KH11	The foot of a mountain overlooking a plain.	Wheat and barley and oil plants such as sunflowers and cotton, in addition to citrus and olive trees, as well as trees on the surface of the mountain such as Thuja, almonds, wild walnuts, oaks and hawthorns. Among the most important flowers are daffodils, anemone, *Rosa davidii*, thyme, Coriander and Tagetes.	https://maps.app.goo.gl/ji8sPh6uEQyvAwhq9
KH12	A village located on a mountainside overlooking a plain with an undulating nature	Natural and cultivated trees, shrubs, and grasses. Among the most important of these plants are the hawthorn trees, the wild plum, the oaks, the willows, the Polygonum aviculare, and the elmleaf blackberry (Rubus ulmifolius), as well as cultivated plants such as beans, lentils, and chickpeas, in addition to anemones, Chamomile, and wild mint.	https://maps.app.goo.gl/VmGbkqkCAKBQeKjy9
KH13	Inside the home garden in the city of Dohuk.	The most important plants in the garden are peach, apricot, grape, Loquat and citrus fruits such as oranges and lemons, as well as vegetables such as eggplant, pepper, okra, and celery.	https://maps.app.goo.gl/KLa3MpUw5SGiHBH49
KH14	Mountainous area with high mountains overlooking the valleys.	Its natural plants are oak, *Pistacia terebinthus*, juniper, Populus, and willow, as well as fruit trees such as apples, plum, grapes, and mulberries. The flowering weeds are hibiscus, irises, and *Fritillaria imperialis*.	https://maps.app.goo.gl/yqLADo3yAWZ5tZ5S8
KH15	Located on the Matin mountain range, the area is covered with natural trees.	Oaks, all kinds of Thuja, and Pirus, Syriaca, as well as shrubs such as wild plum, wild figs, and wild almonds, in addition to fruit trees such as walnuts, almonds, pear, apples, and plum. Its flowering herbs include rosemary, thyme, narcissus, iris, Tagetes, and Althaea.	https://maps.app.goo.gl/9fdR9LRG2AJ9CNfi8
KH16	It is located between mountain valleys.	Pine trees, junipers, wild plum, and thuja, as well as fruit trees such as apples, grapes, pears, walnuts, and almonds, in addition to field crops such as tomatoes, potatoes, radishes, lentils, chickpeas, and beans.	https://maps.app.goo.gl/yMnoQkUqvsacbDP97

Table S2. Additional information on honey samples from Czech and Slovak beekeepers.

Sample	Area	Location	Origin	Production
CZ1	Lipůvka	49°20'18.8"N 16°33'09.2"E	beekeeping	June 2018
CZ2	Vlkaneč	49°48'14.2"N 15°24'05.5"E	beekeeping	May 2018
CZ3	Vlkaneč	49°48'14.2"N 15°24'05.5"E	beekeeping	June 2018
CZ4	Valtice	48°44'34.8"N 16°45'23.8"E	beekeeping	May 2018
CZ5	Brno	49°10'59.3"N 16°36'01.3"E	beekeeping	May 2018
CZ6	Brno	49°10'59.3"N 16°36'01.3"E	beekeeping	June 2018
CZ7	Brno	49°10'59.3"N 16°36'01.3"E	beekeeping	April 2018
CZ8	Brno	49°10'59.3"N 16°36'01.3"E	beekeeping	July 2018
CZ9	Brno	49°10'59.3"N 16°36'01.3"E	beekeeping	May 2018
CZ10	Vlkaneč	49°48'14.2"N 15°24'05.5"E	beekeeping	May 2018
CZ11	Lužice	48°50'09.1"N 17°04'32.2"E	beekeeping	August 2022
CZ12	Lužice	48°50'09.1"N 17°04'32.2"E	beekeeping	July 2022
CZ13	Žeravice	49°01'14.2"N 17°14'06.9"E	beekeeping	June 2022
CZ14	Žeravice	49°01'14.2"N 17°14'06.9"E	beekeeping	August 2022
CZ15	Žeravice	49°01'14.2"N 17°14'06.9"E	beekeeping	July 2022
CZ16	Lužice	48°50'23.8"N 17°04'14.4"E	beekeeping	May 2022
CZ17	Lužice	48°50'23.8"N 17°04'14.4"E	beekeeping	May 2022
CZ18	Trnovec	49°06'52.3"N 17°29'48.7"E	beekeeping	June 2022
CZ19	Mutěnice	48°54'10.9"N 17°01'41.1"E	beekeeping	May 2022
CZ20	Mutěnice	48°54'10.9"N 17°01'41.1"E	beekeeping	May 2022
CZ21	Mutěnice	48°54'10.9"N 17°01'41.1"E	beekeeping	June 2022
CZ22	Lužice	48°50'09.2"N 17°04'32.8"E	beekeeping	July 2022
SK1	Borský Mikuláš	48°37'33.9"N 17°12'20.7"E	beekeeping	May 2018
SK2	Hradište	48°37'48.9"N 17°29'25.6"E	beekeeping	May 2018
SK3	Podbranč	48°44'42.3"N 17°26'50.3"E	beekeeping	June 2018
SK4	Podbranč	48°44'42.3"N 17°26'50.3"E	beekeeping	May 2018
SK5	Kunov	48°42'29.0"N 17°24'43.8"E	beekeeping	June 2018
SK6	Kunov	48°42'29.0"N 17°24'43.8"E	beekeeping	May 2018
SK7	Kunov	48°42'29.0"N 17°24'43.8"E	beekeeping	May 2018
SK8	Kunov	48°42'29.0"N 17°24'43.8"E	beekeeping	April 2018
SK9	Lehota	48°18'42.6"N 17°59'21.2"E	beekeeping	May 2018
SK10	Piešťany	48°36'31.4"N 17°49'22.6"E	beekeeping	May 2018
